# LAPAROSCOPIC ANTIREFLUX SURGERY: WERE OLD QUESTIONS ANSWERED? PARTIAL OR TOTAL FUNDOPLICATION?

**DOI:** 10.1590/0102-672020230023e1741

**Published:** 2023-07-07

**Authors:** Marco Ettore Allaix, Fabrizio Rebecchi, Alex Bellocchia, Mario Morino, Marco Giuseppe Patti

**Affiliations:** 1 University of Torino, Department of Surgical Sciences – Torino, Italy; 2 University of North Carolina at Chapel Hill, Department of Medicine and Surgery – Chapel Hill, United States of America.

**Keywords:** Gastroesophageal reflux, Fundoplication, Laparoscopy, Deglutition disorders, Manometry, Refluxo gastroesofágico, Fundoplicatura, Laparoscopia, Disfagia, Manometria

## Abstract

Laparoscopic total fundoplication is currently considered the gold standard for the surgical treatment of gastroesophageal reflux disease. Short-term outcomes after laparoscopic total fundoplication are excellent, with fast recovery and minimal perioperative morbidity. The symptom relief and reflux control are achieved in about 80 to 90% of patients 10 years after surgery. However, a small but clinically relevant incidence of postoperative dysphagia and gas-related symptoms is reported. Debate still exists about the best antireflux operation; during the last three decades, the surgical outcome of laparoscopic partial fundoplication (anterior or posterior) were compared to those achieved after a laparoscopic total fundoplication. The laparoscopic partial fundoplication, either anterior (180°) or posterior, should be performed only in patients with gastroesophageal reflux disease secondary to scleroderma and impaired esophageal motility, since the laparoscopic total fundoplication would impair esophageal emptying and cause dysphagia.

## RESUMO

A fundoplicatura total laparoscópica é considerada, atualmente, o padrão ouro para o tratamento cirúrgico da doença do refluxo gastroesofágico. Os resultados de curto prazo após a fundoplicatura total laparoscópica são excelentes, com recuperação rápida e morbidade perioperatória mínima. O alívio dos sintomas e o controle do refluxo são alcançados em cerca de 80 a 90% dos pacientes, 10 anos após a cirurgia. No entanto, é relatada uma incidência pequena, mas clinicamente relevante, de disfagia pós-operatória e sintomas relacionados a gases. Ainda existe debate sobre a melhor operação antirrefluxo e, nas últimas três décadas, os resultados cirúrgicos da fundoplicatura parcial laparoscópica (anterior ou posterior) foram comparados aos obtidos após uma fundoplicatura total laparoscópica. A fundoplicatura parcial laparoscópica, seja anterior (180°) ou posterior, deve ser realizada apenas em pacientes com doença do refluxo gastroesofágico secundária a esclerodermia e motilidade esofágica ineficiente, pois uma fundoplicatura total laparoscópica prejudicaria o esvaziamento esofágico e causaria disfagia.

## INTRODUCTION

Laparoscopic total fundoplication (LTF) is considered, today, the procedure of choice for the surgical treatment of gastroesophageal reflux disease (GERD); it increases the resting pressure and the length of the lower esophageal sphincter (LES), decreases the number of transient LES relaxations, and improves quality of esophageal peristalsis^1,2,24^.

An LTF is associated with less morbidity and similar long-term outcomes compared to open fundoplication^6^. The control of symptoms is achieved in about 80–90% of patients 10 years after surgery^10,27^, with similar safety and efficacy in both young and elderly patients^37^. However, LTF is associated with a small but significant incidence of postoperative dysphagia and gas-related symptoms. Several strategies have been proposed to minimize or prevent these adverse effects, including short gastric vessels division during LTF^22,32^, and several variants of LPF (posterior, anterior 90°, anterior 180°).

The best procedure for treating reflux remains an issue of debate^41^. Several randomized clinical trials (RCTs), mostly from Australia, found that an LTF was as effective in controlling reflux as LPF, but it was associated with a higher incidence of postoperative dysphagia and gas-related symptoms^5^. On the other hand, many studies from the United States reported similar rates of postoperative dysphagia after both procedures, with LPF being less effective in controlling reflux than LTF^16,30,33^.

This paper critically reviewed the current evidence about the surgical outcomes of LTF and LPF in the treatment of GERD, comparing LTF to different types of LPF.

## METHODS

Full-text articles in English were selected from searches of the PubMed database (from 1991 to the present) using the following search terms, either alone or in combination:

“gastroesophageal reflux disease”, “laparoscopic”, “total fundoplication”, “partial anterior fundoplication”, “partial posterior fundoplication”, “dysphagia”, “recurrent reflux”, and “gas-bloat syndrome”.

The reference list of all the identified papers was checked for additional articles for inclusion in this review.

## RESULTS

### Laparoscopic fundoplication: total or partial?

A very recently published systematic review and meta-analysis of 26 RCTs and 17 cohort studies by McKinley et al.^25^ tried to answer the question whether an LTF was more effective than LPF in GERD patients. They found no significant differences in early major complications according to Clavien Dindo classification.

The pooled analysis did not reveal any significant differences in terms of symptoms control at both short- and long-term follow-up. Similarly, postoperative objective reflux assessment by pH monitoring showed no differences between LTF and LPF. LPF was associated with prolonged use of proton pump inhibitors (PPIs)^20^. Rates of failure of the wrap and reoperation between laparoscopic total fundoplication (LTF) and laparoscopic partial fundoplication (LPF) were also similar.

A slightly lower risk of long-term (more than 5 years) dysphagia was reported in patients undergoing LPF; however, the difference was not statistically significant. Similarly, there were no different rates in endoscopic dilatation between the two groups of patients.

Lastly, the meta-analysis of RCTs showed similar rates of long-term gas bloating after LPF and LTF. Quality of life was also similar.

One of the major limitations of this meta-analysis is that the authors did not analyze the results according to the type of LPF. We, herein, revised the literature differentiating the types of wrap performed in GERD patients.

#### Anterior 180° Laparoscopic partial fundoplication verus Laparoscopic total fundoplication

Watson et al.^41^ reported in 1999 the short-term results of a prospective double-blind RCT comparing 53 GERD patients undergoing LTF and 54 GERD patients undergoing an anterior 180° LPF. Patients with severe esophageal motility disorder were excluded. Postoperative dysphagia, heartburn, and patient satisfaction were assessed using standardized clinical grading systems. At 6 months, LPF patients experienced significantly less dysphagia for solid food (15% vs. 40%; p=0.008), were more likely to belch normally, reported less flatulence, and their level of satisfaction was higher than patients treated with LTF. No differences were observed in terms of heartburn (9% in both groups), and mean acid exposure at 24-hour pH monitoring. The authors concluded that anterior 180° LPF achieves equivalent control of reflux and was associated with improved clinical outcomes at 6 months.

The 5-year follow-up results of this RCT, based on standardized questionnaires, confirmed in 101 patients (51 LTF and 50 LPF) similar heartburn control in both groups (10% LTF vs. 20% LPF; p=0.172). Besides, we found lower incidence of dysphagia and abdominal bloating, and reduced inability to belch among LPF patients, with high patient satisfaction scores in both groups, proving the durability of anterior 180° LPF^21^.

Finally, a 10-year follow-up data obtained in 89 patients (48 LTF and 41 LPF) using a standard clinical questionnaire showed that both LTF and anterior 180° LPF were durable, safe and effective, with no significant differences in terms of heartburn control, use of PPIs, incidence of dysphagia, and overall satisfaction^8^. However, when patients were tested with manometry and ambulatory 24-hour impedance, pH monitoring at 14-year follow-up, mean LES resting and relaxation pressures were lower and acid, weakly acidic, liquid and mixed reflux episodes were more common after LPF. LPF patients experienced more frequently heartburn than LTF patients, while dysphagia was less common^4^.

Similar results were obtained by Baigrie et al.^1^. They randomized 163 GERD patients, regardless of esophageal motility, to LTF (84 patients) or to anterior 180° LPF (79 patients). They found no significant differences in heartburn according to the assessment by visual analogue scale between the two groups at 3, 12, and 24 months. Patients after LPF had significantly less dysphagia at each follow-up interval. No differences were reported in patient satisfaction scores.

#### Anterior 90° laparoscopic partial fundoplication versus laparoscopic total fundoplication

Although postoperative dysphagia and gas-related problems are reduced after anterior 180° LPF compared to LTF, they are still reported in some patients. This led to the development of an anterior 90° LPF in the late 1990s, that was compared to LTF in several RCTs.

Watson et al.^40^ published in 2004 the short-term outcomes of a multicenter, prospective, double-blind RCT: 112 GERD patients were randomized to anterior 90° LPF (60 patients) or LTF with division of the short gastric vessels (52 patients). Patients with esophageal motility disorders were excluded from the study. Clinical outcomes in terms of dysphagia, heartburn and overall satisfaction were measured using multiple clinical grading systems at 1, 3, and 6 months postoperatively. Esophageal manometry, 24-hour pH monitoring, and upper endoscopy were performed 3 to 4 months after surgery. No significant differences were observed in terms of early postoperative morbidity and length of postoperative stay. At 6 months, dysphagia and flatulence were more frequently experienced by patients undergoing LTF. LES pressure, acid exposure, and endoscopic findings were similar at 3-4 months after both procedures. The incidence of heartburn assessed by yes/no questions was similar in the two groups at 1 and 3 months; however, it was significantly higher after LPF at 6 months (19% vs. 4%; p=0.03). Overall satisfaction was higher after LPF. Based on these data, the authors concluded that anterior 90° LPF provides effective reflux control, and it is followed by less dysphagia and gas-related symptoms than LTF.

A 12-month follow-up of clinical outcome based on analog scales showed that patients after LPF were less likely to experience dysphagia than patients treated with LTF, in contrast, no differences were observed at 5 years. A reduced incidence of heartburn was reported after LTF compared to LPF at 12 months and 5 years. Overall satisfaction was similar in both groups of patients over time^28^.

Spencer et al.^35^ published in 2006 the short-term results of a RCT that compared 40 patients undergoing anterior 90° LPF with 39 patients treated with LTF without division of the short gastric vessels. Patients with severe esophageal motility that contraindicated an LTF were excluded from the study. At 1-year follow-up, LTF was associated with higher rates of dysphagia, while no differences were reported for the assessment of heartburn by the visual analogue scale. However, 24-hour pH monitoring showed a significantly lower percentage time with pH less than 4 in the LTF group. At manometry, postoperative LES resting pressure was similar in both groups, whereas LES residual relaxation pressure was significantly higher after LTF.

In a study, 74 patients were available for analysis of clinical outcome using standardized questionnaires at 5 years^39^. The incidence of dysphagia and gas bloating was higher after LTF when measured by an analogue score. There were no significant differences in terms of heartburn control and overall satisfaction, although PPIs were more frequently used after LPF (29.7% vs. 8.1%). However, manometry and pH monitoring were not performed.

Broeders et al.^7^ combined raw data sets from these two RCTs, and used the original data to determine the clinical outcomes at 5-year follow-up. Data were available from a subset of 90 patients undergoing LPF and 82 patients treated with LTF. Heartburn scores were significantly higher after LPF, and the use of PPIs was more common. In this group of patients, however, dysphagia and gas-related symptoms were less frequent. Overall satisfaction with the surgical outcomes were similar. No differences were observed in terms of endoscopic dilatations performed for dysphagia (2 vs. 6%; p=0.202), and the number of reoperations (10 vs. 4.9%; p=0.212). In particular, most frequent indication for reoperation was recurrent reflux in the LPF group, and dysphagia in the LTF group.

These data can be summarized as follows (Table 1):

- Both 180° and 90° anterior LPF were associated with less postoperative dysphagia than LTF at 5-year follow-up. However, at 10 years after surgery, the outcome following anterior 180° LPF and LTF were not significantly different.
- At 5 years, incidence of reflux symptoms (i.e., heartburn) and use of PPIs after anterior 180° LPF and LTF were similar, but higher after anterior 90° LPF than LTF.
- Recurrent reflux was the most common indication for surgical revision of an anterior LPF, while persistent dysphagia was the leading cause for reoperation after LTF. However, the overall number of surgical revisions was not significantly different for both LPF and LTF.
- Overall patient satisfaction rating was similar after both subtypes of anterior LPF and LTF.

**Table 1:** Surgical outcomes after laparoscopic anterior partial fundoplication and laparoscopic total fundoplication.

Reference	Fundoplication	n	Follow-up	Heartburn	Acid exposure	Dysphagia	Quality of evidence
Watson, et al.^41^	Anterior 180°	54	6 months	Partial=LTF	Partial=LTF	Partial<LTF	Moderate
	LTF	53					
Ludemann, et al.^21^	Anterior 180°	50	5 years	Partial = LTF	NP	Partial<LTF	Moderate
	LTF	51					
Cai, et al.^8^	Anterior 180°	41	10 years	Partial = LTF	NP	Partial=LTF	Moderate
	LTF	48					
Broeders, et al.^6^ *	Anterior 180°	36	14 years	Partial > LTF	Partial > LTF	Partial<LTF	Moderate
	LTF	41					
Baigrie, et al.^1^	Anterior 180°	79	2 years	Partial = LTF	NP	Partial<LTF	Moderate
	LTF	84					
Watson, et al.^40^	Anterior 90°	60	6 months	Partial>LTF	Partial=LTF	Partial<LTF	Moderate
	LTF	52					
Watson, et al.^39^	Anterior 90°	53	5 years	Partial>LTF	NP	Partial=LTF	Moderate
	LTF	44					
Spencer, et al.^35^	Anterior 90°	40	1 year	Partial=LTF	Partial > LTF	Partial<LTF	Moderate
	LTF	39					
Watson, et al.^39^	Anterior 90°	37	5 years	Partial=LTF	NP	Partial<LTF	Moderate
	LTF	37					
Broeders, et al.^5^ **	Anterior 90°	90	5 years	Partial>LTF	NP	Partial<LTF	Moderate
	LTF	81					

LTF: laparoscopic total fundoplication; NP: not performed.

* performed in 8 LPF and 10 LTF;

** combined analysis of references 7 and 18.

However, these results should be interpreted with caution. Indeed, most RCTs included small number of patients, did not perform 24-hour pH monitoring to evaluate the incidence of reflux at long-term follow-up, and considered symptom control and the use of PPIs as a marker of surgical outcome. In fact, many studies showed that when ambulatory 24-hour pH monitoring was performed to test patients with recurrent heartburn, pathological reflux was present in less than 40% of cases^12,18,38^. On the other hand, long-term studies showed a less effective control of gastroesophageal reflux with a LPF^2,29,33^. Recurrence of gastroesophageal reflux confirmed by pH monitoring at 5 years is reported in more than 50% of patients after LPF^16,30,33^.

Based on these data, LTF should be considered, today, the leading treatment option for GERD in patients with normal esophageal motility.

#### Posterior laparoscopic partial fundoplication versus laparoscopic total fundoplication

Laparoscopic posterior fundoplication has been proposed as an alternative to LTF in order to reduce the incidence of postoperative dysphagia and gas-related symptoms in GERD patients with normal esophageal peristalsis^15^. Several large RCTs were published, but the results of these studies showed no significant differences and did not allow to draw any definitive conclusion. Broeders et al.^5^ published in 2010 a systematic review and meta-analysis of RCTs comparing LTF to Toupet fundoplication (posterior partial) for GERD, aiming to establish the best surgical procedure, according to the highest level of evidence. They identified seven RCTs comparing 404 LTF patients and 388 Toupet patients^19,34^. The methodological quality of the included RCTs ranged from poor to excellent, with a median Jadad score of 2 (range 1–5). Follow-up ranged between 12 months (4 RCTs) and 60 months (1 RCT). LTF was associated with a significantly higher prevalence of dysphagia, inability to belch and gas bloating after surgery, more endoscopic dilatations and more surgical reoperations. No differences were observed for recurrent pathological acid exposure, esophagitis, reflux symptoms, and overall patient satisfaction.

These data can be summarized as follows (Table 2):

- Toupet fundoplication and LTF achieved similar reflux control.
- Toupet fundoplication was associated with reduced postoperative dysphagia, need for endoscopic dilatation, reoperation rates, and prevalence of gas-related symptoms compared to LTF. However, these initial mechanical advantages seemed to disappear over time^23^.

**Table 2:** Surgical outcomes after laparoscopic posterior partial fundoplication (Toupet) and laparoscopic total fundoplication.

Reference	Fundoplication	n	Follow-up (months)	Heartburn	Acid exposure	Dysphagia	Quality of evidence
Booth, et al.^3^	Toupet	63	12	Toupet=LTF	Toupet=LTF	Toupet=LTF	Low
	LTF	64					
Chrysos, et al.^9^	Toupet	19	12	NA	NA	Toupet=LTF	Low
	LTF	14					
Guérin, et al.^13^	Toupet	63	12	Toupet=LTF	NA	Toupet=LTF	Low
	LTF	77					
Laws, et al.^19^	Toupet	16	27	Toupet=LTF	NA	Toupet=LTF	Low
	LTF	23					
Mickevicius, et al.^26^	Toupet	77	12	Toupet=LTF	NA	Toupet=LTF	Low
	LTF	76					
Shaw, et al.^34^	Toupet	50	60	Toupet=LTF	Toupet=LTF	Toupet=LTF	High
	LTF	50					
Strate, et al.^36^	Toupet	100	24	Toupet=LTF	Toupet>LTF	Toupet <LTF	Low
	LTF	100					
Broeders, et al.^5^ *	Toupet	388		Toupet = LTF	Toupet=LTF	Toupet<LTF	Low
	LTF	404					

Toupet: laparoscopic posterior partial fundoplication; LTF: laparoscopic total fundoplication; NA: not available.

* meta-analysis of the RCTs included in the table.

This meta-analysis presents some major limitations:

- Heterogenous methodological quality of the RCTs included in the study.
- Different indications for surgery (GERD proven on 24-hour pH monitoring, GERD proven on upper endoscopy, GERD requiring daily PPI therapy).
- Short-follow-up.
- Small number of patients enrolled in each RCT.
- Lack of objective evaluation of heartburn by 24-hour pH monitoring after antireflux surgery.

A longer period of more than 5 years of data follow-up is required to confirm similar long-term outcomes after Toupet and LTF, since several large prospective and retrospective studies suggested that Toupet fundoplication results in poorer long-term reflux control. For instance, Jobe et al.^17^ found in 100 consecutive GERD patients that 24-hour pH monitoring was abnormal in 51% of all patients and in 39% of asymptomatic patients after laparoscopic Toupet fundoplication. Similarly, Patti et al.^31–33^ found that at 70 months after surgery in 56% of patients after laparoscopic posterior fundoplication, but only 28% after LTF had persistent reflux confirmed by 24-hour pH monitoring. After posterior fundoplication, more patients took PPIs (25 vs. 8%) or required a second operation (9 vs. 3%). The incidence of postoperative dysphagia was similar in the two groups, showing that the type of fundoplication (total or partial) is not a risk factor for dysphagia.

Based on these data, we consider that an LTF is today the procedure of choice for the treatment of GERD in patients with normal esophageal motility.

#### Laparoscopic partial fundoplication: anterior or posterior?

Based on the similar reflux control and reduced postoperative dysphagia after LPF reported in several RCTs, few studies investigated the surgical outcomes of different partial fundoplications. Hagedorn et al.^14^ looked at the efficacy and mechanical consequences in 95 GERD patients who were randomized to have an anterior 120° LPF (47 patients) or a posterior (Toupet) LPF (48 patients). At 12 months, both procedures were effective in reducing reflux symptoms. However, significantly fewer patients experienced postoperative heartburn and regurgitation after a posterior LPF. Similarly, significant differences were observed in 24-hour pH monitoring in favor of posterior LPF: even if acid exposure was reduced after both operations, normal levels were achieved only after a posterior LPF. No significant differences between the two groups were recorded in terms of postoperative dysphagia and ability to belch.

At 5 years, the long-term results of this RCT showed that a posterior LPF provided significantly better heartburn and regurgitation control, with lower number of reoperations and use of PPIs^11^.

These data can be summarized as follows:

- Posterior LPF achieves better reflux control, with no increase in postoperative dysphagia at short- and long-term follow-up. However, further RCTs with long-term follow-up are needed to confirm these results.

Based on these limited data, we consider that a posterior LPF is superior to an anterior LPF.

## CONCLUSIONS

LTF is today the procedure of choice for the treatment of GERD patients. The LPF, either anterior (180°) or posterior, should be performed only in patients with GERD secondary to scleroderma and impaired esophageal motility, since an LTF would impair esophageal emptying and cause dysphagia.
